# Neoadjuvant radiotherapy improves overall survival for T3/4N+M0 rectal cancer patients: a population-based study of 20300 patients

**DOI:** 10.1186/s13014-020-01497-4

**Published:** 2020-02-27

**Authors:** Feng Zhao, Jili Wang, Hao Yu, Xiaofei Cheng, Xinke Li, Xuan Zhu, Xiangming Xu, Jianjiang Lin, Xin Chen, Senxiang Yan

**Affiliations:** 1 Department of Radiation Oncology, The First Affiliated Hospital, College of Medicine, Zhejiang University, Hangzhou, Zhejiang 310003 People’s Republic of China; 2 Graduate School, College of Medicine, Zhejiang University, Hangzhou, Zhejiang 310003 People’s Republic of China; 3 Department of Colorectal Surgery, The First Affiliated Hospital, College of Medicine, Zhejiang University, Hangzhou, Zhejiang 310003 People’s Republic of China; 4 Institute of Pharmaceutical Biotechnology and The First Affiliated Hospital, College of Medicine, Zhejiang University, Hangzhou, Zhejiang 310058 People’s Republic of China

**Keywords:** Rectal cancer, Radiotherapy (RT), Surgery, Chemotherapy, Overall survival (OS)

## Abstract

**Background:**

Neoadjuvant radiotherapy (RT) has been shown to improve local control; however, whether it can improve overall survival (OS) in locally advanced rectal cancer (LARC) patients remains controversial. We therefore aimed to examine the benefits of surgery alone, neoadjuvant radiotherapy (RT), adjuvant RT, and surgery plus chemotherapy in stage II (T3/4N0M0) and III (any T and N + M0) on the OS of rectal cancer patients.

**Methods:**

Date from the Surveillance, Epidemiology, and End Results (SEER) database diagnosed between 2004 and 2016 were used. Kaplan-Meier analyses were used to compare patient prognoses across different treatment modalities. Cox hazard regression analysis were used to identify independent predictors of OS.

**Results:**

For stage T3/4N0M0 patients, neoadjuvant RT, adjuvant RT, and surgery plus chemotherapy resulted in similar OS (all *p* > 0.05; mean survival, 115.89 months (M), 111.97 M, and 117.22 M, respectively), with better OS observed in these patients than in patients who underwent surgery alone (all *p* < 0.001, mean survival, 88.96 M). For stage T1/2N + M0 patients, neoadjuvant RT, adjuvant RT, and surgery plus chemotherapy resulted in similar OS (all *p* > 0.05; mean survival, 121.50 M, 124.25 M, and 121.20 M, respectively), with better OS observed in these patients than in patients who underwent surgery alone (all *p* < 0.001, mean survival 83.81 M). For stage T3/4N + M0 patients, neoadjuvant RT (HR = 0.436; 95% CI, 0.396~0.478; *p* < 0.001) resulted in significantly longer OS than adjuvant RT and surgery plus chemotherapy (mean survival, 104.47 M, 93.94 M, and 93.62 M, respectively), with better OS observed in these patients than in patients who underwent surgery alone (all *p* < 0.001, mean survival 54.87 M). Older age (> 60 years), black race, unmarried status, high tumour grade, and tumour size > 5 cm were all associated with a poor prognosis (all *p* < 0.05).

**Conclusions:**

Neoadjuvant RT, adjuvant RT, and surgery plus chemotherapy results in better OS than surgery alone in LARC patients. Neoadjuvant RT has the potential to be highly recommended over adjuvant RT and surgery plus chemotherapy for T3/4N + M0 patients; however, it showed no OS advantage over adjuvant RT or surgery plus chemotherapy for T3/4N0M0 and T1/2N + M0 patients.

## Background

Colorectal cancer (CRC) is the third most commonly diagnosed cancer among both men and women in the United States, and one-third of CRC cases are rectal in origin [1]. Due to the close proximity of the rectum to pelvic structures and organs, the absence of a serous membrane surrounding the rectum, and the restriction of the surgical view and access by the pelvic cavity, the locoregional recurrence (LRR) rate in rectal cancer is relatively high after surgery alone [2]. As previously, without adjuvant therapy, the locoregional recurrence (LRR) rate of stage II (T3 or T4 and N0) and III (N+) rectal cancer was 15 to 65% [3].

To improve local control after conventional surgery, radiotherapy (RT) has been utilized. In the 1980s and 1990s, several randomized controlled trials (RCTs) showed that preoperative or postoperative RT could reduce the LRR rate and improve the overall survival (OS) rate, laying the foundation for the comprehensive treatment mode of surgery combined with RT instead of surgery alone [4–8]. However, at the time of these studies, the concept of total mesorectal excision (TME) for rectal cancer was not widely accepted, so neoadjuvant RT not only reduced LRR rate but also improved the OS rate. The modern era of rectal cancer surgery started with the introduction of TME by Heald and Ryall in 1986 [9, 10]. In the era of TME, a Dutch trial from the Netherlands reported that TME combined with RT reduced LRR without increasing OS compared to TME alone, after a median follow-up of 12 years [11]. Therefore, although most trials have shown consistent benefits in the risk of local relapse in stage II and III rectal cancer, controversy still exists regarding the OS benefit of TME combined with RT.

In addition, several studies have compared the preoperative administration of RT (neoadjuvant RT) versus the postoperative administration of RT (adjuvant RT) for stage II and III rectal cancer. A prospective randomized trial from the German Rectal Cancer Study Group (the CAO/ARO/AIO-94 trial) compared preoperative versus postoperative chemoradiotherapy (CRT) for the treatment of clinical stage II and III rectal cancer, and showed that preoperative RT was associated with a significant reduction in local recurrence and treatment-associated toxicity; however, OS was similar in the two groups [12]. Consequently, the National Cancer Comprehensive Network (NCCN) guidelines have adopted preoperative CRT as the standard of care for stage II–III rectal cancer [3]. However, whether preoperative CRT is superior to postoperative CRT for OS remains unclear. Meanwhile, several pilot studies suggested that preoperative chemotherapy combined with TME instead of preoperative CRT plus TME because surgery combined with RT did not show any benefits in terms of OS and elicitedseveral adverse side effects related to RT [13, 14].

Because of conflicting survival data and the lack of population level data, we sought to examine the following using the Surveillance, Epidemiology, and End Results (SEER) Program of the National Cancer Institute: 1) patient demographics, and general clinical characteristics of stage II and III rectal cancer; and 2) OS of surgery alone, neoadjuvant RT, adjuvant RT, and surgery plus chemotherapy for stage II and III rectal cancer patients. Although, the treatment details (i.e., surgical margins, radiation dose, chemotherapy regimen and chemotherapy sequence) and some clinical information (distance from the anal verge, tumour markers and lateral lymph nodes) are not recorded in the SEER database, this database covers a sampling of approximately 26% of the United States (US) population, which is considered representative of the US patients in terms of its demographic composition, cancer incidence and mortality.

## Methods

### Study population

This study was based on a secondary analysis of previously collected, publicly available and de-identified data. The SEER database holds no identifying patient information, all data are anonymous, and therefore, written informed consent was not needed for this study. This investigation was conducted in accordance with the ethical standards of the Declaration of Helsinki and with national and international guidelines. The institutional review board of our hospital approved this study.

The cohort used to estimate the patient demographics and survival was created using SEER 18 Registries Custom Data (with additional treatment fields), with a November 2018 Submission (1973–2016 varying).

**Fig. 1:**
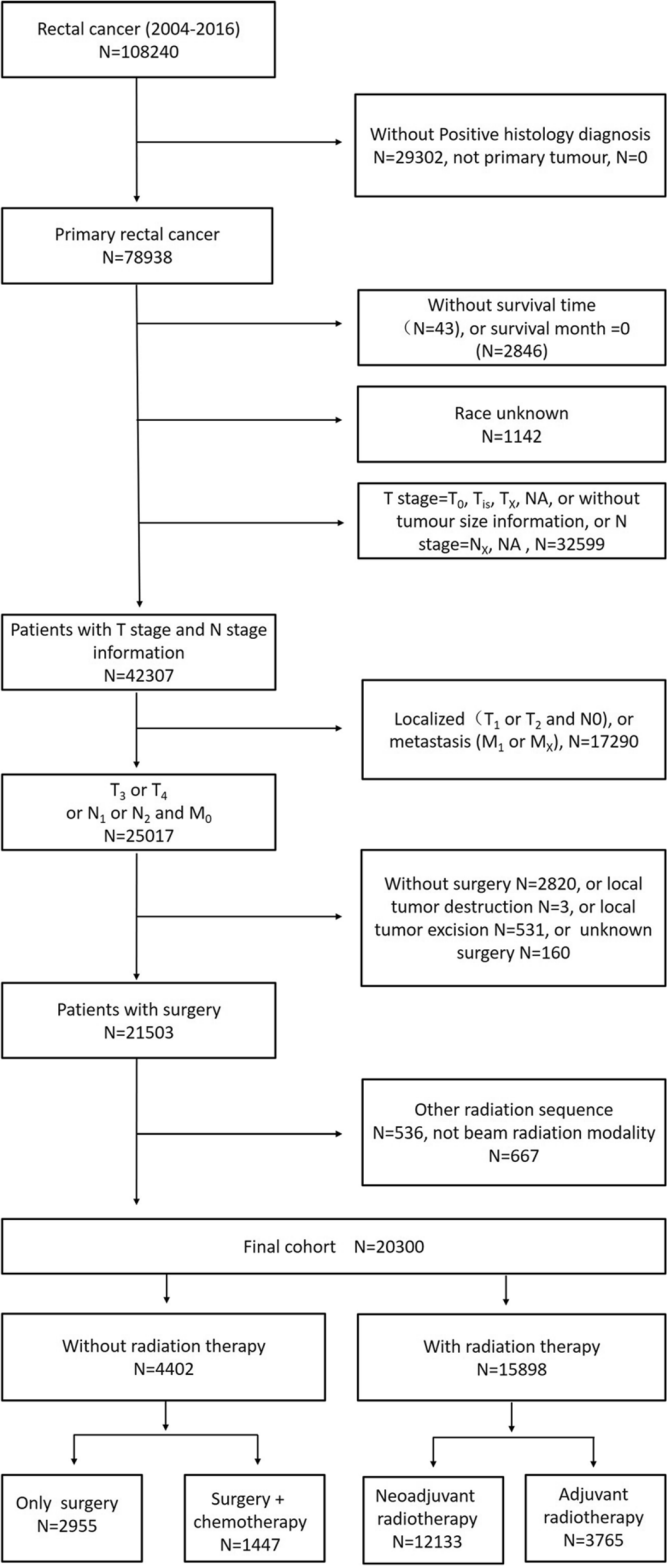
Flow chart for the creation of the patient cohort data set

Rectal cancer diagnosed between 2004 and 2016 by histologic confirmation either from biopsy or surgical pathology, rather than by clinical presentation, radiography, autopsy, or death records alone, were selected. In addition, we included only patients with tumour sequence numbers labelled “one primary only” and with follow-up information, tumour size information and racial information. The cohort was restricted to locally advanced rectal cancer (LARC: T3 or T4 with any N and M0; and any T with N1 or N2 and M0). After the exclusion of patients without surgery (or unknown surgery or local tumour excision only) and the restriction of the radiation sequence to “no radiation”, “radiation after surgery” and “radiation prior to surgery”, as well as the restriction of the radiation code to “beam radiation” and “none/unknown”, 20,300 patients were included in the study (Fig. 1). Patients were stratified into the following 4 groups on the basis of treatment strategies: 1. Only surgery group patients who received surgery alone without radiation therapy or chemotherapy; 2. Surgery + chemotherapy group patients who received surgery plus chemotherapy (neoadjuvant or adjuvant chemotherapy) without radiation therapy; 3. Neoadjuvant radiotherapy group patients who received neoadjuvant radiation therapy plus surgery with or without chemotherapy; and 4. Adjuvant radiotherapy group patients who received surgery plus adjuvant radiation therapy with or without chemotherapy.

### Variable definitions

Covariates of interest that were extracted for each case included patient demographic variables (age at diagnosis, sex, race, and marital status), tumour characteristics (tumour grade, American Joint Committee on cancer stage (AJCC stage, 6th edition and the SEER-derived combined stage 2016) and TNM stage (jointly determined by AJCC stage, 6th edition, and the SEER-derived combined stage 2016)), and treatment modality (surgery, RT, and chemotherapy).

### Statistical analyses

Categorical data were compared using the Chi-square test. Survival probabilities were estimated using the Kaplan-Meier method, and the log-rank test was used to assess any significant differences in OS, which was stratified by each covariate. Cox proportional hazards models were used to analyse associations of patient characteristics and treatment modalities with patient survival. Only variables that were significantly associated with survival in the univariate Cox analysis were included in the multivariate Cox analysis. Hazard ratios (HRs) and 95% confidence intervals (CIs) were estimated using univariate and multivariable models. Statistical analysis was performed with SPSS version 25.0 (SPSS Inc., Chicago, IL, USA), and *p* < 0.05 was considered statistically significant.

## Results

### Patient characteristics

Demographic data for LARC patients is shown in Table 1. The mean age at diagnosis was 60.76 ± 13.207 years. Most of the patients were male, white, and married. The majority of patients (71.2%) had moderately differentiated tumours. Among the 20,300 included patients, 7808 (38.5%) and 12,492 (61.5%) were categorized as stage II and stage III, respectively. Regarding treatment, 14.6% of patients were managed with surgery alone, 59.8% received neoadjuvant RT prior to surgery, and 18.5% received adjuvant RT after surgery.

**Table 1:** Patient demographics and clinical characteristics (*n* = 20300)

Characteristics	Level	Number (%)
Age at diagnosis, years	Mean ± SD	60.76 ± 13.207
	Median (range)	60 (17~101)
	≤60	10283(50.7%)
	> 60	10017(49.3%)
Sex	Male	12097(59.6%)
	Female	8203(40.4%)
Race	White	16403(80.8%)
	Black	1663(8.2%)
	American Indian/Alaska Native	166(0.8%)
	Asian or Pacific Islander	2068(10.2%)
Marital status	Married	11834(58.3%)
	Unmarried	7742(38.1%)
	Unknown	724(3.6%)
Tumour grade	Well differentiated	1255(6.2%)
	Moderately differentiated	14457(71.2%)
	Poorly differentiated	2676(13.2%)
	Undifferentiated	293(1.4%)
	Unknown	1616(8.0%)
Tumour size	0~3 cm	5856(28.8%)
	3~5 cm	8097(39.9%)
	> 5 cm	6347(31.3%)
AJCC Stage	II	7808(38.5%)
	III	12492(61.5%)
AJCC T Stage	T1	554(2.7%)
	T2	1609(7.9%)
	T3	16223(79.9%)
	T4	1914(9.4%)
AJCC N Stage	N0	7808(38.5%)
	N1	8745(43.1%)
	N2	3747(18.5%)
Treatment modality	Only surgery	2955(14.6%)
	Neoadjuvant RT	12133(59.8%)
	Adjuvant RT	3765(18.5%)
	Surgery + chemotherapy	1447(7.1%)

*RT* radiotherapy

According to the stratification by treatment modality (Table 2), in the cohort of surgery alone, the mean age at diagnosis was 70.94 ± 13.68 years, 76.0% patients were older than 60 years, and more than half of patients (54.1%) were stage II. In the cohorts of neoadjuvant and adjuvant RT, most of the patients (62.2 and 58.5% respectively) were male, and the majority of patients (98.0 and 91.5% respectively) also received chemotherapy. In the cohort of surgery plus chemotherapy, the majority of patients were stage III.

**Table 2:** Patient characteristics stratified by treatment modality (*n* = 20300)

Characteristics	Level	Only surgery (*n* = 2955)	Surgery + chemotherapy (*n* = 1447)	Neoadjuvant RT (*n* = 12,133)	Adjuvant RT (*n* = 3765)	*P* value
Age, years	Mean ± SD	70.94 ± 13.680	59.60 ± 12.824	58.56 ± 12.305	60.28 ± 12.037	–
	Median (range)	73 (20~101)	59 (17~92)	58 (17~99)	60 (21~95)	
	≤60	708 (24.0%)	764 (52.8%)	6880 (56.7%)	1931 (51.3%)	< 0.001
	> 60	2247 (76.0%)	683 (47.2%)	5253 (43.3%)	1834 (48.7%)	
Sex	Male	1556 (52.7%)	793 (54.8%)	7547 (62.2%)	2201 (58.5%)	< 0.001
	Female	1399 (47.3%)	654 (45.2%)	4586 (37.8%)	1564 (41.5%)	
Race	White	2387 (80.8%)	1161 (80.2%)	9827 (81.0%)	3028 (80.4%)	0.027
	Black	233 (7.9%)	121 (8.4%)	992 (8.2%)	317 (8.4%)	
	American Indian /Alaska Native	9 (0.3%)	8 (0.6%)	117 (1.0%)	32 (0.8%)	
	Asian or Pacific Islander	326 (11.0%)	157 (10.9%)	1197 (9.9%)	388 (10.3%)	
Marital status	Married	1408 (47.6%)	882 (61.0%)	7221 (59.5%)	2323 (61.7%)	< 0.001
	Unmarried	1401 (47.4%)	504 (34.8%)	4495 (37.0%)	1342 (35.6%)	
	Unknown	146 (4.9%)	61 (4.2%)	417 (3.4%)	100 (2.7%)	
Tumour grade	Low	2423 (82.0%)	1163 (80.4%)	9149 (75.4%)	2977 (79.1%)	< 0.001
	High	495 (16.8%)	250 (17.3%)	1537 (12.7%)	687 (18.2%)	
	Unknown	37 (1.3%)	34 (2.3%)	1447 (11.9%)	101 (2.7%)	
Tumour size	0~3 cm	654 (22.1%)	442 (30.5%)	3657 (30.1%)	1103 (29.3%)	< 0.001
	3~5 cm	1260 (42.6%)	577 (39.9%)	4752 (39.2%)	1508 (40.1%)	
	> 5 cm	1041 (35.2%)	428 (29.6%)	3724 (30.7%)	1154 (30.7%)	
AJCC Stage	II	1598 (54.1%)	343 (23.7%)	4653 (38.3%)	1214 (32.2%)	< 0.001
	III	1357 (45.9%)	1104 (76.3%)	7480 (61.7%)	2551 (67.8%)	
AJCC T Stage	T1	93 (3.1%)	102 (7.0%)	168 (1.4%)	191 (5.1%)	< 0.001
	T2	240 (8.1%)	203 (14.0%)	699 (5.8%)	467 (12.4%)	
	T3	2395 (81.0%)	1006 (69.5%)	10,057 (82.9%)	2765 (73.4%)	
	T4	227 (7.7%)	136 (9.4%)	1209 (10.0%)	342 (9.1%)	
AJCC N Stage	N0	1598 (54.1%)	343 (23.7%)	4653 (38.3%)	1214 (32.2%)	< 0.001
	N1	889 (30.1%)	717 (49.6%)	5598 (46.1%)	1541 (40.9%)	
	N2	468 (15.8%)	387 (26.7%)	1882 (15.5%)	1010 (26.8%)	
Chemotherapy	Yes	0 (0.0%)	1447 (100.0%)	11,890 (98.0%)	3445 (91.5%)	< 0.001
	No/Unknown	2955 (100.0%)	0 (0.0%)	243 (2.0%)	320 (8.5%)	

*RT* radiotherapy. Low: well differentiated and moderately differentiated; High: Poorly differentiated and undifferentiated

### Patient survival

**Fig. 2:**
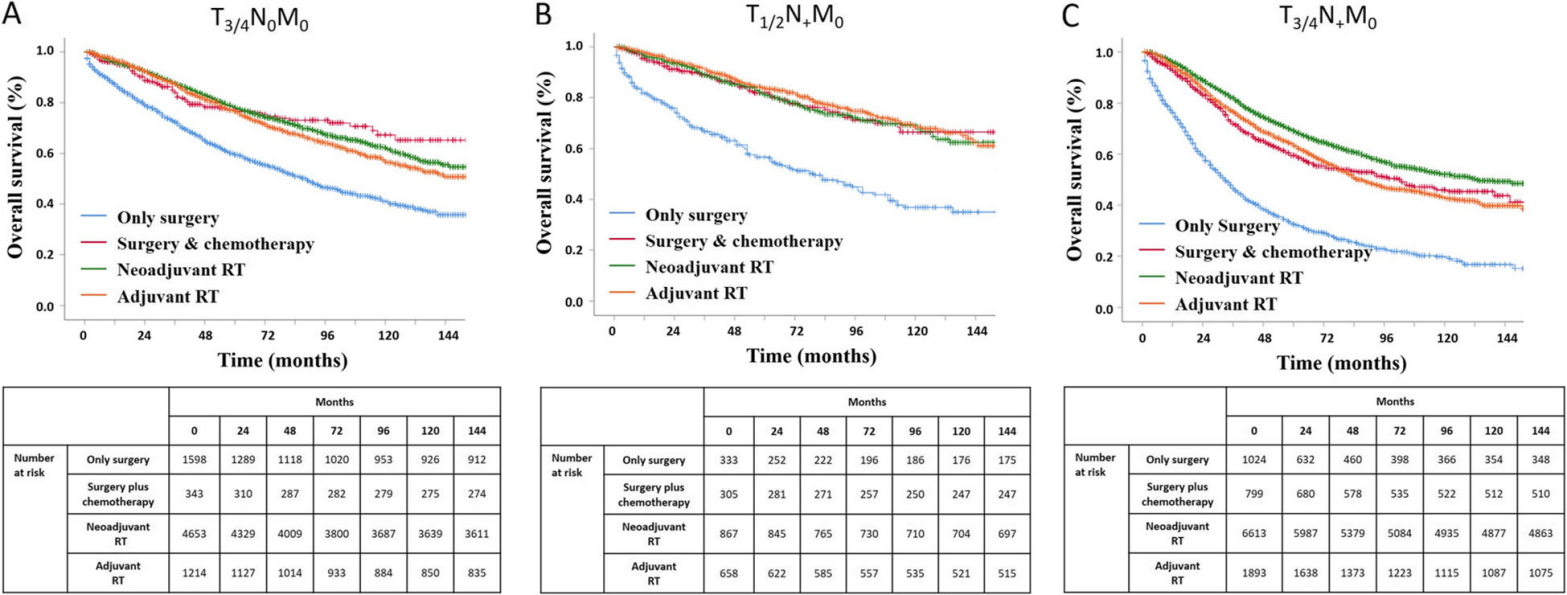
OS estimated with the Kaplan-Meier method for stage II and III rectal cancer. **a** OS estimated with the Kaplan-Meier method for patients with T_3/4_N_0_M_0_ stage disease receiving different treatment modalities (surgery alone versus (vs.) adjuvant RT: *p* < 0.001; surgery alone vs. neoadjuvant RT: *p* < 0.001; surgery alone vs. surgery plus chemotherapy: *p* < 0.001; adjuvant RT vs. neoadjuvant RT: *p* = 0.051; adjuvant RT vs. surgery plus chemotherapy: *p* = 0.214; and neoadjuvant RT vs. surgery plus chemotherapy: *p* = 0.724). **b** OS estimated with the Kaplan-Meier method for patients with T_1/2_N_+_M_0_ stage disease receiving different treatment modalities (surgery alone vs. adjuvant RT: *p* < 0.001; surgery alone vs. neoadjuvant RT: *p* < 0.001; surgery alone vs. surgery plus chemotherapy: *p* < 0.001; adjuvant RT vs. neoadjuvant RT: *p* = 0.332; adjuvant RT vs. surgery plus chemotherapy: *p* = 0.442; and neoadjuvant RT vs. surgery plus chemotherapy: *p* = 0.906); **c** OS estimated with the Kaplan-Meier method for patients with T_3/4_N_+_M_0_ stage disease receiving different treatment modalities (surgery alone vs. adjuvant RT: *p* < 0.001; surgery alone vs. neoadjuvant RT: *p* < 0.001; surgery alone vs. surgery plus chemotherapy: *p* < 0.001; adjuvant RT vs. neoadjuvant RT: *p* < 0.001; adjuvant RT vs. surgery plus chemotherapy: *p* = 0.637; and neoadjuvant RT vs. surgery plus chemotherapy: *p* < 0.001)

Kaplan-Meier curves for the OS of rectal cancer patients are shown in Fig. 2. The mean, 2-, 5-, and 10-year survival of rectal cancer patients are shown in Table 3. After adjusting for age, sex, race, marital status, tumour grade, tumour size and N stage, multivariate Cox analyses of different treatment modalities were performed, and the results for OS are shown in Table 4. For stage II and III rectal cancer patients, neoadjuvant RT, adjuvant RT, and surgery plus chemotherapy resulted in significantly longer OS than surgery alone (all *p* < 0.001). Specifically, for stage T3/4N0M0, neoadjuvant RT (HR = 0.591; 95% CI, 0.533–0.654; *p* < 0.001), adjuvant RT (HR = 0.660; 95% CI, 0.581–0.749; *p* < 0.001), and surgery plus chemotherapy (HR = 0.598; 95% CI, 0.466–0.768; *p* < 0.001) had similar OS outcomes (all *p* > 0.05), with a mean survival of 115.89 months (M), 111.97 M, and 117.22 M, respectively. For stage T1/2N + M0 patients, neoadjuvant RT (HR = 0.467; 95% CI, 0.372–0.587; *p* < 0.001), adjuvant RT (HR = 0.378; 95% CI, 0.299–0.477; *p* < 0.001), and surgery plus chemotherapy (HR = 0.463; 95% CI, 0.342–0.628; *p* < 0.001) also had similar OS outcomes (all *p* > 0.05), with a mean survival of 121.50 M, 124.25 M, and 121.20 M, respectively. For stage T3/4N + M0 patients, neoadjuvant RT (HR = 0.436, 95% CI, 0.396~0.478; *p* < 0.001) had significantly better OS outcomes than adjuvant RT (HR = 0.478; 95% CI, 0.431–0.531; *p* < 0.001) and surgery plus chemotherapy (HR = 0.495; 95% CI, 0.431–0.568; *p* < 0.001), with mean survival of 104.47 M, 93.94 M, and 93.62 M, respectively.

**Table 3:** Mean survival and, 2-, 5-, and 10-year OS of rectal cancer patients (*n* = 20300)

Variables		Mean survival	2-year OS	5-year OS	10-year OS
T_3/4_N_0_M_0_	Only surgery	88.96 M	71.9%	59..6%	41.1%
	Neoadjuvant RT	115.89 M	92.3%	78.1%	62.2%
	Adjuvant RT	111.97 M	92.4%	76.7%	56.8%
	Surgery + chemo	117.22 M	88.7%	77.3%	67.3%
T_1/2_N_+_M_0_	Only surgery	83.81 M	74.3%	56.7%	36.8%
	Neoadjuvant RT	121.50 M	93.4%	81.1%	69.3%
	Adjuvant RT	124.25 M	94.2%	83.6%	68.6%
	Surgery + chemo	121.20 M	91.2%	81.9%	66.6%
T_3/4_N_+_M_0_	Only surgery	54.87 M	59.3%	32.5%	19.4%
	Neoadjuvant RT	104.47 M	88.9%	69.2%	52.0%
	Adjuvant RT	93.94 M	85.6%	63.5%	42.8%
	Surgery + chemo	93.62 M	83.1%	59.6%	46.0%

*OS* overall survival, *RT* radiotherapy

**Table 4:** Multivariate Cox analyses of treatment modalities for OS (*n* = 20300)

	Treatment	Multivariate	*P* value
		HR (95% CI)	
T_3/4_N_0_M_0_ (*n* = 7808)	Surgery alone	1	
	Neoadjuvant RT	0.591 (0.533~0.654)	< 0.001
	Adjuvant RT	0.660 (0.581~0.749)	< 0.001
	Surgery + chemo	0.598 (0.466~0.768)	< 0.001
T_1/2_N_+_M_0_ (*n* = 2163)	Surgery alone	1	
	Neoadjuvant RT	0.467 (0.372~0.587)	< 0.001
	Adjuvant RT	0.378 (0.299~0.477)	< 0.001
	Surgery + chemo	0.463 (0.342~0.628)	< 0.001
T_3/4_N_+_M_0_ (*n* = 10,329)	Surgery alone	1	
	Neoadjuvant RT	0.436 (0.396~0.478)	< 0.001
	Adjuvant RT	0.478 (0.431~0.531)	< 0.001
	Surgery + chemo	0.495 (0.431~0.568)	< 0.001

Multivariate Cox analyses are adjusted by age, sex, race, marital status, tumour grade, tumour size and N stage; *HR* Hazard ratio, *RT* radiotherapy

Using univariate and multivariate Cox proportional hazards analysis (Table 5), older age, black race, unmarried status, high tumour grade, and tumour size > 5 cm were all associated with a poor prognosis (all *p* < 0.001).

**Table 5:** Univariate and multivariate analyses for OS of all patients (*n* = 20300)

Variables	Level	Univariate	*P* value	Multivariate	*P* value
		HR (95% CI)		HR (95% CI)	
Age	≤60	1		1	
	> 60	2.196(2.084~2.313)	< 0.001	2.177(2.067~2.294)	< 0.001
Sex	Male	1			
	Female	0.966(0.918~1.016)	0.179		
Race	White	1		1	
	Black	1.231(1.131~1.340)	< 0.001	1.226(1.126~1.335)	< 0.001
	American Indian/Alaska Native	1.112(0.846~1.461)	0.447	1.213(0.923~1.595)	0.166
	Asian or Pacific Islander	0.859(0.787~0.939)	0.001	0.922(0.844~1.007)	0.071
Marital status	Married	1		1	
	Unmarried	1.553(1.476~1.633)	< 0.001	1.456(1.384~1.533)	< 0.001
	Unknown	1.186(1.027~1.369)	0.020	1.142(0.989~1.320)	0.070
Tumour grade	Low	1		1	
	High	1.623(1.527~1.726)	< 0.001	1.646(1.548~1.750)	< 0.001
	Unknown	0.788(0.705~0.880)	< 0.001	0.807(0.722~0.902)	< 0.001
Tumour size	0~3 cm	1		1	
	3~5 cm	1.186(1.114~1.263)	< 0.001	1.164(1.094~1.240)	< 0.001
	> 5 cm	1.443(1.353~1.539)	< 0.001	1.399(1.311~1.493)	< 0.001

*OS* overall survival, *HR* Hazard ratio

## Discussion

### Older age at diagnosis and unmarried status were associated with poor OS

According to the demographic data in our study, the median age at diagnosis of LARC patients was 60 years, which agrees with a previous study by Rolf Sauer, et al. [15]. Meanwhile, we found that older age (> 60 years) significantly increased the risk of mortality, and in the cohort of surgery alone, the median age at diagnosis was 73 years, which agrees with a study by Peng et al. [16]. In our study, approximately 60% of patients were male, which agrees with previous studies [11, 17, 18]. Marital status has been increasingly recognized as an important factor in the survival of cancer patients [19]. In our study, most patients were married (58.3%), and we found that being married was associated with better patient outcomes. Meanwhile, in the cohorts of neoadjuvant RT, adjuvant RT, and surgery plus chemotherapy, approximately 60% of the patients were married, while only approximately 50% of patients in the surgery alone group was married. This phenomenon was possibly due to married patients receiving social and financial support from their families, which tended to lead to the choice of a proactive treatment modality.

### Black ethnic background was associated with poor OS

In this study, white patients accounted for the largest proportion of patients (80.8%), which was consistent with the race distribution in the Western population [20]. Our study demonstrated that black race is a significant risk factor for OS, similar to a former study [16]. One possible reason for this finding is that black men are less likely to be treated with curative intent than are white men. Interestingly, our study demonstrated that the Asian or Pacific Islander ethnics background might reduce the risk of poor prognosis, with marginal significance (HR = 0.922; 95% CI, 0.844–1.007; *p* = 0.071). Similarly, Zhang et al. reported that Asians achieved better survival rates than other races or patients with locally advanced colon cancer [21]. Differences in diet and other lifestyle factors may affect survival.

### High tumour grade and large tumour size were associated with poor OS

Our study indicated that high pathological grades of the tumour was associated with a worse OS. This finding is in line with the results of a former study [16]. Additionally, we found that tumour size > 5 cm was associated with a poor prognosis, which agrees with the results of a previous study by Kornprat et al., which demonstrated that a median tumour size of colorectal cancer exceeding 4.5 cm was significantly associated with survival [22]. A meaningful cut-off value for the prediction of the progression of rectal cancer patients could be further studied.

### Neoadjuvant RT was the most frequent strategy for LARC

Regarding treatment modalities, 59.8% of patients received neoadjuvant RT, which is currently the most popular strategy for LARC patients, which is in line with the NCCN guidelines indicating that preoperative CRT serves as the standard of care for stage II–III rectal cancer [3]. In addition, both short-course radiotherapy (25 Gy in 5 fractions) and long-course radiotherapy (50.4 Gy in 28 fractions, conventionally fractionated therapy) can be applied as neoadjuvant radiotherapy [3]. However, short-course RT is only a concept and rarely used in US. Therefore, the overwhelming majority of patients probably has likely received the conventionally fractionated therapy in our study.

### LARC patients received only surgery were associated with poor OS

In the pre-TME era, several RCTs have indicated that neoadjuvant or adjuvant RT could reduce the LRR rate and improve the OS [4–8]. In the TME era, in the majority of randomized trials of chemotherapy and/or radiotherapy, no OS benefits have been observed, despite the marked improvement in local control rates [23]. The Dutch trial also reported that short-term RT plus TME did not increase OS compared to TME alone in all pooled resectable rectal cancer patients [11]. However, in the Dutch trial, for the patients with TNM stage III cancer with a negative circumferential resection margin, neoadjuvant RT plus TME group had better 10-year OS than the only TME group [11]. In our study, the vast majority of patients in the only surgery group were treated with TME since the patients included were diagnosed from 2004 to 2016, a period during the TME era. Our study demonstrated that LARC patients who received only surgery exhibited worse OS than patients who received neoadjuvant RT plus surgery, surgery plus adjuvant RT, and surgery plus chemotherapy. It is worth noting, however, that the average age of the patients who received only surgery was approximately 70 years, higher than that of the other groups, which was approximately 60 years. Older age (> 60 years) was also associated with a poor prognosis. Therefore, whether neoadjuvant RT plus TME, or TME plus adjuvant RT, TME plus chemotherapy is better than surgery alone in LARC patients remains unclear and warrants further investigation.

### Neoadjuvant RT improves OS for T3/4N + M0 rectal cancer patients

The main purpose of this study is to determine whether neoadjuvant RT is superior to postoperative RT in OS prognosis, which is still controversial based on the existing evidence. The recognized advantages of neoadjuvant RT over adjuvant RT are primarily related to tumour response and to the preservation of normal tissue and include the following: 1. downstaging of the tumour, which is conducive to surgical resection and increases the probability of sphincter preservation; 2. increased RT sensitivity because of the better oxygenation of the pelvic tissue before surgery; and 3. avoidance of irradiation of the small bowel, which remains trapped in the pelvis after surgery [2]. To date, however, only several randomized trials have compared the OS benefits between neoadjuvant RT and adjuvant RT for stage II and III patients. Among them, Park JH et al., followed 240 stage II and III patients and reported that a significant benefit was not demonstrated for preoperative CRT in local control and survival [24]. The CAO/ARO/AIO-94 trial followed 799 stage II and III patients and demonstrated that there was a persistent significant improvement in local control for pre- versus postoperative CRT; but there was no effect on OS [15, 25]. However, the NSABP R-03 trial included 267 stage II and III patients, and demonstrated that preoperative CRT, compared with postoperative CRT, significantly improved disease-free survival (DFS) and showed a trend towards improving OS [26]. In this study, we showed that neoadjuvant RT contributed to prolonging the OS compared with adjuvant RT only in the group of stage T3/4N + M0 patients with a mean survival of 104.47 M versus 93.94 M. Interestingly, it was noted that in stage T1/2N + M0 patients, adjuvant RT showed a trend towards improving OS compared to that of neoadjuvant RT, with a mean survival of 124.25 M versus 121.5 M, with only marginal significance. Additionally, Peng et al. reported that adjuvant RT had better 10-year CSS than neoadjuvant RT for T3N0M0 patients [16]. Therefore, the former randomized trials may not have shown significant OS benefits of neoadjuvant RT due to the lack of a subgroup analysis of patients with stage T3/4N0M0, T1/2N + M0, and T3/4N + M0, and the OS benefits for T3/4N + M0 patients might have been weakened by the other groups.

Several studies have evaluated the effectiveness of the addition of concurrent chemotherapy to neoadjuvant or adjuvant RT. A meta-analysis reported that neoadjuvant RT improves local control in patients with rectal cancer, particularly when CRT is administered [27]. Additionally, a Cochrane review of 6 RCTs reported that the addition of chemotherapy to neoadjuvant RT in stage III patients reduced the risk of LRR but had no benefits for OS [28]. Furthermore, EORTC 22941 and FFCD 9203 tested different combinations of RT and chemotherapy and demonstrated that 5-FU-based chemotherapy combined with RT has the best results [29, 30]. In addition, several studies demonstrated that total neoadjuvant therapy (TNT), in which chemoradiation and chemotherapy are administered prior to surgery, is a viable treatment for LARC [31–33]. In our study, the majority of patients who underwent neoadjuvant RT (98.0%) and adjuvant RT (91.5%), also received chemotherapy. However, the chemotherapy information from the SEER database was limited and lacked information on the specific chemotherapy protocols and chemotherapy sequences, which warrants further investigation.

Recently, a treatment strategy involving neoadjuvant chemotherapy (a kind of surgery plus chemotherapy strategy) has been proposed by several studies, because the majority of previous studies demonstrated that RT has several side effects, such as bowel dysfunction, urinary toxicity, sexual dysfunction, and even secondary malignancies after RT [14, 34–36]; additionally RT has no benefit for OS [11, 12, 15, 37]. Schrag et al. demonstrated that for selected patients with clinical stage II to III rectal cancer, neoadjuvant chemotherapy and selective radiation do not seem to compromise outcomes [13]. Very recently, Eisterer et al. reported that neoadjuvant chemotherapy with bevacizumab, capecitabine and oxaliplatin followed by concomitant standard chemoradiation is feasible for patients with LARC and results in a complete pathologic remission (pCR) rate of 25% and a neoadjuvant chemotherapy completion rate of 80% [38]. In our study, in stage T3/4N0M0 and T1/2N + M0 rectal cancer patients, the effect of surgery plus chemotherapy was similar to that of neoadjuvant RT and of adjuvant RT in regard to the increase in OS. However, due to the limitations of the SEER database information, our study failed to analyse the role of neoadjuvant chemotherapy in patients and further research is needed.

### Limitations

Similar to other studies that have utilized the SEER database as their data source, our study has limitations that demand precautious interpretation of the results. First, although the SEER data include information regarding the use of surgery, RT, and chemotherapy, the details of these therapies (i.e., surgical margins, radiation dose, chemotherapy regimen and chemotherapy sequence) are not recorded in the database. Second, the SEER database lacks some key clinical information that might be important for prognosis, such as the distance from the anal verge, tumour markers, extramural vascular invasion (EMVI), lateral lymph nodes, and so on. Third, although this study was conducted in the era of TME, the SEER database did not provide TME information, and we excluded patients who underwent local tumour destruction or excision. Fourth, the SEER database lacks the local control information needed to analyse the correlation between local control and OS, which could help us understand the impact of local control on survival.

## Conclusions

In conclusion, this retrospective study analysed cases in the SEER database from 2004 to 2016 and suggests the following: 1) For stage II and III rectal cancer patients, neoadjuvant RT, adjuvant RT, and surgery plus chemotherapy had a longer OS than surgery alone. 2) For T3/4N0M0 and T1/2N + M0 patients, there was no significant difference in OS among the treatment modalities of neoadjuvant RT, adjuvant RT, and surgery plus chemotherapy. 3) For T3/4N + M0 patients, neoadjuvant RT had significantly longer OS than adjuvant RT and surgery plus chemotherapy. and 4) Older age (> 60 years), black race, unmarried status, high tumour grade, and tumour size > 5 cm were associated with a poor prognosis (all *p* < 0.05). While RCTs should be conducted to confirm these results, it may be appropriate for guidelines to adopt a more proactive stance on using of neoadjuvant RT for T3/4N + M0 rectal cancer patients.

## Data Availability

The data that support the findings of this study are openly available in Surveillance, Epidemiology, and End Results (SEER) database of the National Cancer Institute at https://seer.cancer.gov/.

## Abbreviations

AJCC: American Joint Committee on cancer
CIs: Confidence intervals
CRC: Colorectal cancer
CRT: Chemoradiotherapy
DFS: Disease-free survival
EMVI: Extramural vascular invasion
HRs: Hazard ratios
LRR: Locoregional recurrence
NCCN: National Cancer Comprehensive Network
OS: Overall survival
pCR: complete pathologic remission
RCTs: Randomized controlled trials
RT: Radiotherapy
SEER: Surveillance, Epidemiology, and End Results
TME: Total mesorectal excision
TNT: Total neoadjuvant therapy; US: United States
